# Excessive Supraventricular Ectopic Activity and the Risk of Atrial Fibrillation and Stroke: A Systematic Review and Meta-Analysis

**DOI:** 10.3390/jcdd9120461

**Published:** 2022-12-15

**Authors:** Min Yang, Yapeng Lin, Hang Cheng, Danni Zheng, Song Tan, Liping Zhu, Zimeng Li, Xiaoyun Wang, Jie Yang

**Affiliations:** 1 Department of Neurology, The First Affiliated Hospital of Chengdu Medical College, Chengdu 610500, China; 2 International Clinical Research Center, Chengdu Medical College, Chengdu 610500, China; 3 Biomedical Informatics and Digital Health, School of Medical Sciences, University of Sydney, Sydney, NSW 2050, Australia; 4 Department of Neurology, Sichuan Provincial People’s Hospital, University of Electronic Science and Technology of China, Chengdu 610072, China; 5 Department of Neurology, Affiliated Drum Tower Hospital of Nanjing University Medical School, Nanjing 210008, China

**Keywords:** stroke, excessive supraventricular ectopic activity, atrial fibrillation, mortality, meta-analysis

## Abstract

Background: Excessive supraventricular ectopic activity (ESVEA) is correlated with the development of atrial fibrillation (AF) and is frequently observed in ischemic stroke patients. This meta-analysis aims to summarize the evidence on the association between ESVEA and the risk of AF and stroke. Methods: PubMed and Embase databases were systematically searched to identify all publications providing relevant data from inception to 23 August 2022. Hazard ratio (HR) and 95% confidence interval (CI) were pooled using fixed-effect or random-effect models. Results: We included 23,272 participants from 20 studies. Pooled results showed that ESVEA was associated with an increased risk of AF in the general population (HR: 2.57; 95% CI 2.16–3.05), increased risk of AF in ischemic stroke patients (HR: 2.91; 95% CI 1.80–4.69), new-onset ischemic stroke (HR: 1.91; 95% CI 1.30–2.79), and all-cause mortality (HR: 1.41; 95% CI 1.24–1.59). Pooled analysis indicated that ESVEA was not associated with recurrent ischemic stroke/transient ischemic attack (TIA) (HR: 1.24; 95% CI 0.91–1.67). Conclusions: ESVEA is associated with AF, new-onset ischemic stroke, and all-cause mortality.

## 1. Introduction

Each year, approximately 795,000 people experience a new or recurrent stroke. Of all strokes, 87% are ischemic strokes [1]. Cryptogenic ischemic strokes (or strokes of unknown cause) are thought to comprise about 25% of all ischemic strokes, and most cryptogenic strokes are thromboembolic [2]. Atrial fibrillation (AF) is the most common cardiac arrhythmia and is an independent risk factor for stroke [3]. Meanwhile, AF is associated with increased in-hospital mortality, not only in ischemic stroke patients [4,5,6,7] but also in patients with cardioembolic stroke [8]. It has also been reported that AF is a predictor of early embolic recurrence in patients with cardioembolic stroke [9] and that early recurrent embolization is the most important predictor of in-hospital mortality [10]. Accumulating studies demonstrated a close relationship between premature atrial contractions (PACs) and AF [11,12,13,14,15,16,17,18,19,20]. Moreover, PACs are common in the general population [21] and patients with ischemic stroke [22]. The term ‘excessive supraventricular ectopic activity’ (ESVEA), with varying definitions, has been used to describe different manifestations of excessive atrial ectopic beats in previous studies, which was defined as >30 PACs per hour and/or runs of ≥20 PACs [12,23], PAC/h > 4 and/or supraventricular runs of >5 beats [24], or >100 PACs per 24 h [13,25]. Therefore, ESVEA has been interpreted as a combination of frequent PACs (the number of PACs/h) and/or frequent atrial tachycardia (the number of continuous PACs in any episode). Recently, more observational studies have indicated associations between ESVEA and AF, stroke, and mortality [11,13,15,26,27,28,29,30].

This meta-analysis aims to summarize the evidence on the associations between ESVEA and AF, stroke, and mortality. Recognizing the risk of stroke after ESVEA is vital for informing early primary and secondary stroke prevention.

## 2. Materials and Methods

The meta-analysis was conducted in compliance with the Preferred Reporting Items for Systematic Reviews and Meta-Analyses (PRISMA) guidelines [31]. The study protocol was registered in the International Prospective Register of Systematic Reviews (PROSPERO), ID CRD42022353287. As we reviewed only previously published data, local institutional review board or ethics committee approval and subjects′ informed consent were not required.

### 2.1. Study Search

PubMed and Embase were searched from inception to 23 August 2022 by 2 reviewers (MY and HC). Variations of the following search terms were used: stroke, atrial premature complexes, and atrial fibrillation. The complete search strategy is provided in Supplementary Materials. The reference lists of eligible articles were also scrutinized to find additional data sources.

### 2.2. Study Selection

Two investigators (MY and HC) independently searched the titles and abstracts for articles relevant to our systematic review. If a decision could not be made based on the information in the title and abstract, then the full text was reviewed. Studies were included if they met the following criteria: (1) reported PAC as a risk factor for AF and/or stroke in participants age ≥ 18 years; (2) prospective or retrospective cohort study; (3) follow-up period ≥ 6 months; (4) PAC was detected using ECG or other cardiac telemetry methods, and PAC burden was greater than the presence of PAC; (5) AF and/or stroke were reported as outcome events; (6) the hazard ratio (HR) and the corresponding 95% confidence interval (CI) were reported. The exclusion criteria were as follows: (1) patients with a known history of AF; (2) patients with a history of catheter ablation, percutaneous coronary intervention, or coronary artery bypass graft; (3) patients with implantable cardiac monitoring. There were no language restrictions.

### 2.3. Data Extraction and Quality Assessment

The two investigators independently extracted data from the included studies using prepared forms. The extracted information included the name of the first author, year of publication, country, study design, study population, number of subjects, age, sex, the definition of ESVEA, methods of ESVEA detection, the prevalence of ESVEA, outcomes, and number of interesting outcomes. MY and HC assessed the quality of the included studies according to the Newcastle–Ottawa Scale (NOS) [32]. Any disagreements were resolved by a third author (JY).

### 2.4. Outcomes

The primary outcomes were AF and stroke (including new-onset and recurrent stroke), and the secondary outcome was all-cause mortality.

### 2.5. Statistical Analysis

We pooled the adjusted effect estimates to investigate the independent relationship between ESVEA and the outcomes of AF, stroke, or mortality. The pooled effect estimates were presented as HRs and 95% CIs using the random-effects model (if the heterogeneity was obvious I^2^ statistics > 50%); otherwise, the fixed-effects model was adopted. The Egger test was used, and a funnel plot was constructed to evaluate publication bias (*p*-value < 0.05 was considered significant). Statistical analysis was performed with Stata, version 15.

## 3. Results

**Figure 1:**
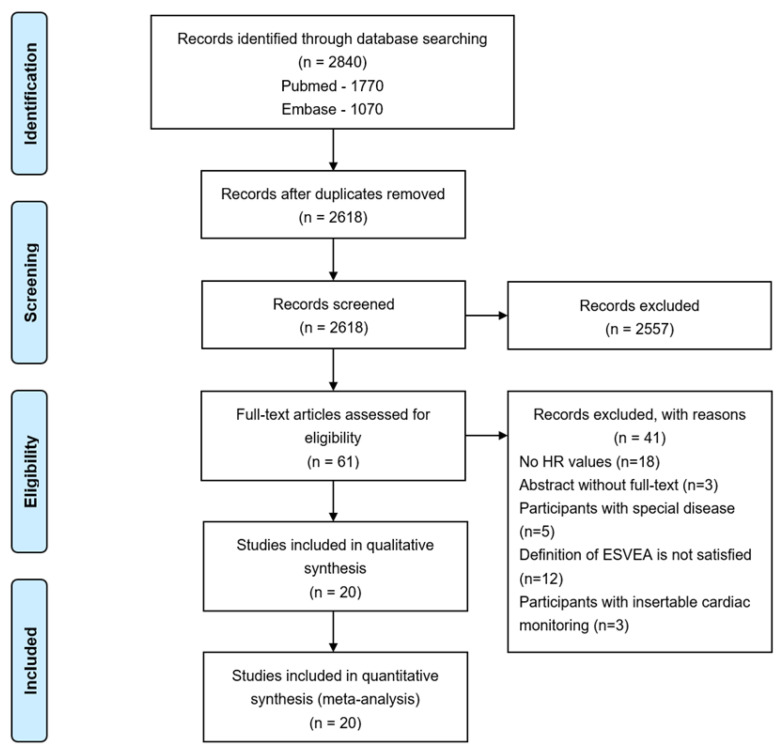
PRISMA flowchart. Abbreviations: HR—hazard ratio; ESVEA—excessive supraventricular ectopic activity.

In total, 2840 records were retrieved through database searching, of which 222 were duplicates. After screening titles and abstracts, 61 articles were included for full-text review. Finally, 20 studies that satisfied all eligibility criteria were included in the review. A detailed flowchart of the screening process is presented in Figure 1.

### 3.1. Characteristics of the Included Studies

In total, 20 studies involving 23,272 participants were included, and the characteristics of the included studies are shown in Table 1 and Table 2. Of the 20 studies, 13 were prospective cohort studies [12,13,14,23,33,34,35,36,37,38,39,40,41] and 7 were retrospective cohort studies [15,22,42,43,44,45,46]. The majority of studies were conducted in Europe (n = 13) [12,22,23,34,36,38,42,44,45], followed by Asia (n = 4) [13,33,43,46] and America (n = 3) [14,15,37]. The number of participants at baseline ranged from 68 [33] to 6100 [45]. The studies used different measurement methods to determine ESVEA: 24 h ECG was used in 13 studies [13,14,15,33,34,35,36,38,41,42,43,44,46], 48 h ECG was used in 4 studies [12,22,23,40], routine ECG was used in 1 study [39], 30 s ECG was used in 1 study [45], and polysomnogram-based ECG was used in 1 study [37]. No studies were graded as having low-quality scores (<5 on the NOS) (Table S1).

### 3.2. Association between ESVEA and AF

#### 3.2.1. Association between ESVEA and AF in the General Population

**Figure 2:**
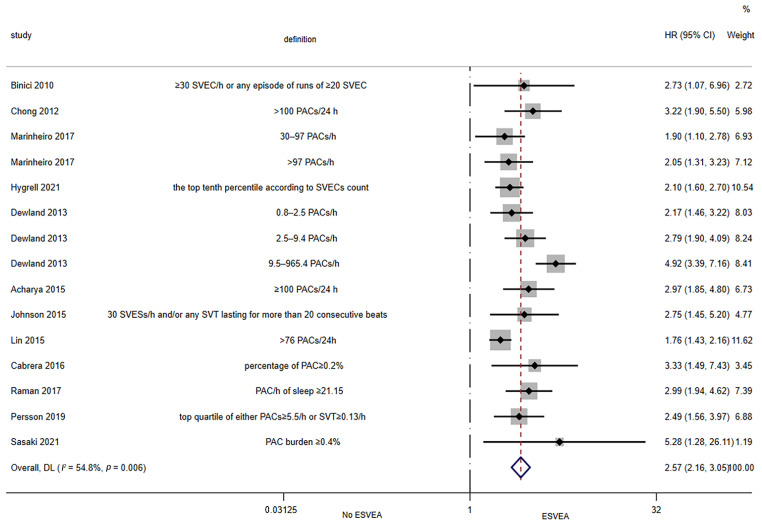
Forest plot of the association between ESVEA and AF in the general population. Abbreviations: SVEC—supraventricular ectopic complexes; PACs—premature atrial complexes; SVESs—supraventricular extrasystoles; SVT—supraventricular tachycardias; ESVEA—excessive supraventricular ectopic activity; HR—hazard ratio; CI—confidence interval. Definition represents the definitions of ESVEA in different studies [12,13,14,15,34,35,37,38,43,44,45,46].

Twelve studies assessed the association between ESVEA and the risk of AF in the general population [12,13,14,15,34,35,37,38,43,44,45,46]. The random-effects pooled adjusted HR was 2.57 (95% CI 2.16–3.05; I^2^ = 54.8%, *p* = 0.006), indicating that ESVEA increased the risk of AF in the general population (Figure 2). The results of the Egger’s tests (*p* = 0.039) and funnel plot (Figure S1) showed publication bias.

#### 3.2.2. Association between ESVEA and AF in the Ischemic Stroke Patients

**Figure 3:**
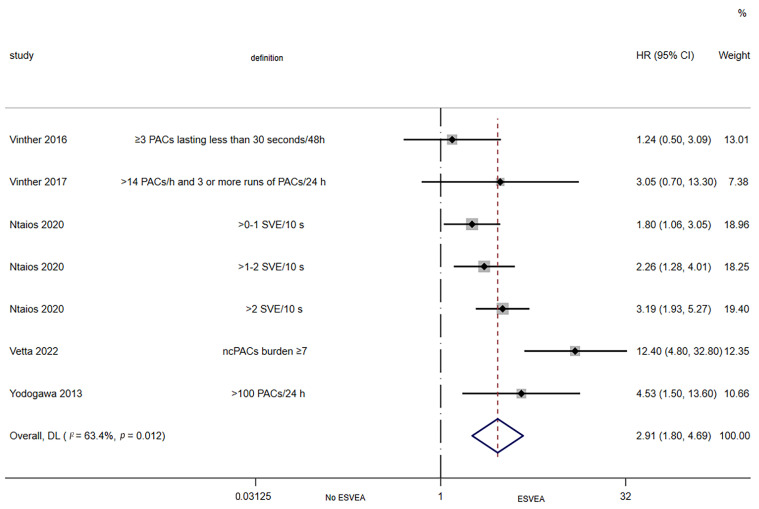
Forest plot of the association between ESVEA and AF in the ischemic stroke patients. Abbreviations: PACs—premature atrial complexes; SVE—supraventricular extrasystoles; ncPACs—non-conducted premature atrial complexes; ESVEA—excessive supraventricular ectopic activity; HR—hazard ratio; CI—confidence interval. Definition represents the definitions of ESVEA in different studies [22,33,36,39,41].

Five studies assessed the association between ESVEA and the risk of AF in ischemic stroke patients [22,33,36,39,41]. The random-effects pooled adjusted HR was 2.91 (95% CI 1.80–4.69; I^2^ = 63.4%, *p* = 0.012), indicating that ESVEA increased the risk of AF in ischemic stroke patients (Figure 3).

### 3.3. Association between ESVEA and Stroke

#### 3.3.1. Association between ESVEA and Risk of New-Onset Ischemic Stroke

**Figure 4:**
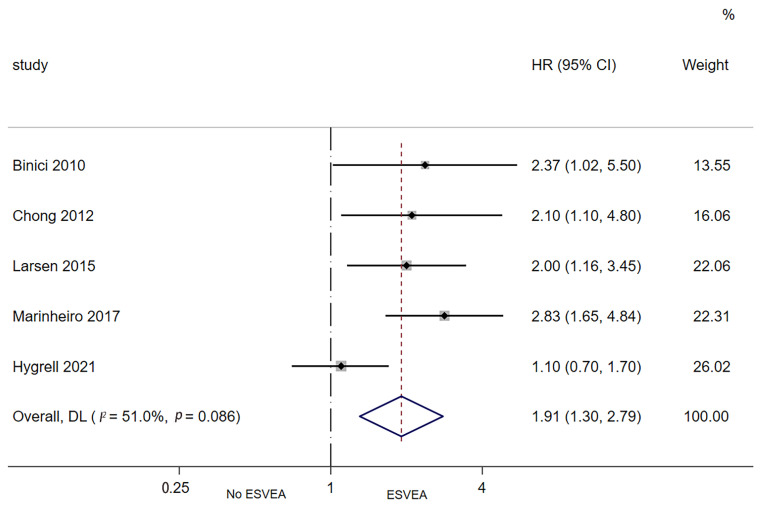
Forest plot of the association between ESVEA and new-onset ischemic stroke. Abbreviations: ESVEA—excessive supraventricular ectopic activity; HR—hazard ratio; CI—confidence interval [12,13,23,35,45].

Five studies assessed the association between ESVEA and the risk of new-onset ischemic stroke [12,13,23,35,45]. The random-effects pooled adjusted HR was 1.91 (95% CI 1.30–2.79; I^2^ = 51%, *p* = 0.086), indicating that ESVEA increased the risk of new-onset ischemic stroke (Figure 4).

#### 3.3.2. Association between ESVEA and Risk of Recurrent Ischemic Stroke/Transient Ischemic Attack (TIA)

**Figure 5:**
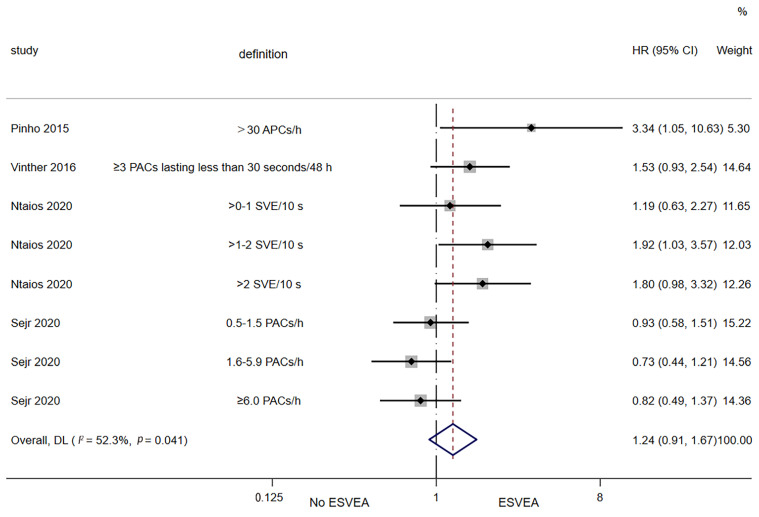
Forest plot of ESVEA and the risk of recurrent ischemic stroke/transient ischemic attack. Abbreviations: APCs—atrial premature complexes; PACs—premature atrial complexes; SVE—supraventricular extrasystoles; ESVEA—excessive supraventricular ectopic activity; HR—hazard ratio; CI—confidence interval. Definition represents the definitions of ESVEA in different studies [22,39,40,42].

Four studies assessed the association between ESVEA and the risk of recurrent ischemic stroke/TIA [22,39,40,42]. The random-effects pooled adjusted HR was 1.24 (95% CI 0.91–1.67; I^2^ = 52.3%, *p* = 0.041), indicating that ESVEA did not increase the risk of recurrent ischemic stroke/TIA (Figure 5).

### 3.4. Association between ESVEA and All-Cause Mortality

**Figure 6:**
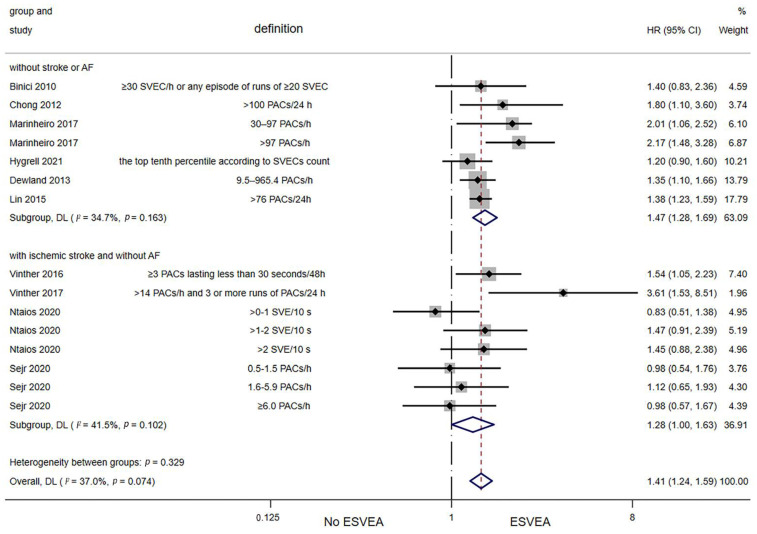
Forest plot of the association between ESVEA and all-cause mortality. Abbreviations: SVEC—supraventricular ectopic complexes; PACs—premature atrial complexes; SVE—supraventricular extrasystoles; ESVEA—excessive supraventricular ectopic activity; HR—hazard ratio; CI—confidence interval. Definition represents the definitions of ESVEA in different studies [12,13,14,22,35,36,39,40,43,45].

Ten studies assessed the association between ESVEA and the risk of all-cause mortality [12,13,14,22,35,36,39,40,43,45]. The random-effects pooled adjusted HR was 1.41 (95% CI 1.24–1.59; I^2^ = 37%, *p* = 0.074), indicating that ESVEA increased the risk of all-cause mortality (Figure 6). The results of the Egger′s test (*p* = 0.655) and funnel plot (Figure S2) demonstrated a lack of publication bias.

## 4. Discussion

In this systematic review and meta-analysis, we summarized the relationship between ESVEA and AF, stroke, and death. Pooled data showed that ESVEA had more than doubled the risk of AF in the general population from twelve studies and in ischemic stroke patients from five studies. Pooled data based on five studies showed that ESVEA was correlated with a nearly two-fold increase in the risk of new-onset ischemic stroke. Ten studies indicated that ESVEA increases more than doubled the risk of all-cause mortality.

### 4.1. Thoughts on Antithrombotic Therapy of ESVEA

Our results indicated that ESVEA increased the risk of AF and new-onset stroke. According to the guidelines, anticoagulation in patients with AF depends on the CHA2DS2-VASc score [47]. However, the current guidelines do not recommend antiplatelet or anticoagulant therapy in patients with ESVEA. Whether non-stroke patients with ESVEA would benefit from antithrombotic therapy is still unclear. Future trials on the primary prevention of stroke using antiplatelet therapy for patients with ESVEA are warranted. Before these trials are carried out, high-quality studies are urgently needed to verify the relationship between ESVEA and the risk of new-onset stroke. In addition, our analysis indicated that ESVEA did not increase the risk of recurrent ischemic stroke/TIA, which may be due to the use of antiplatelets in 62% of patients after their first stroke [48]. Thus, antiplatelet therapy may be an effective secondary prevention treatment for stroke patients with ESVEA, and further studies are needed to prove this hypothesis.

### 4.2. Definition of ESVEA

At present, the term ‘ESVEA’ has various definitions from different studies, mainly because the cut-off value for the word ‘excessive’ is not clear. For example, Binici et al. [12] had set the cut-off value at the top 10th percentile for both frequency of supraventricular ectopic complexes (SVEC) and length of runs of SVEC, so they defined ESVEA as ≥30 SVEC per hour or any episode of runs of ≥20 SVEC. Weber-Krüger et al. [24] had set the cut-off value at the median of PAC frequency and the longest supraventricular run on 24 h-Holter (SV-run 24 h), so they defined ESVEA as PAC/h >4 and longest SV-run 24 h >5. In the future, larger and more standardized studies are urgently needed to unify the cut-off values and definitions of ESVEA. Thus, we could better diagnose ESVEA among patients, with a view toward early AF or stroke prevention. 

### 4.3. Detection Methods of ESVEA

Furthermore, we found that the methods for detecting ESVEA were different in the included studies, including routine ECG, 24 h ECG, 48 h ECG, and polysomnogram-based ECG. The American Heart Association/American Stroke Association guideline for the prevention of stroke recommends that prolonged rhythm monitoring (≈30 days) is reasonable within six months of the index event for ischemic stroke or transient ischemic attack (TIA) patients with no other apparent cause [47]. Some studies have indicated the use of prolonged, continuous ECG monitoring for the detection of undiagnosed AF in stroke/TIA patients [49,50]. We need large sample studies with appropriate ECG monitoring methods and adequate follow-up duration to verify that ESVEA increases the risk of AF and stroke in the future. 

### 4.4. Limitations

There are several limitations in the present systematic review and meta-analysis. Firstly, the pooled results were highly heterogeneous; we conducted subgroup and sensitivity analyses to find the source of heterogeneity in these studies. When different ECG durations were analyzed as subgroups, the heterogeneity of pooled outcomes between ESVEA and risk of new-onset and recurrent stroke were significantly reduced (Figure S3, Figure S4). Although our results indicated that ESVEA increased the risk of AF in the general population, the Egger′s test and funnel plot showed publication bias. Large samples and high-quality studies are needed to confirm the results in the future. Secondly, all included studies were observational, stroke subtypes and the duration of follow-up were variable, the detection method and definition of ESVEA were different, and the influence of confounders could not be fully excluded. Thirdly, we could not include any randomized controlled trials or large prospective studies in our analysis due to the lack of such studies, which could affect the reliability of our results.

### 4.5. Future Research

We advocate further investigation of the underlying mechanisms of ESVEA. For example, Bayés syndrome is associated with a high incidence of atrial tachyarrhythmias, which could be the cause of delayed and retrograde activation of the left atrium [51,52]. Bayés syndrome has also been shown to be a predictor of cardioembolic stroke [53,54,55]. Furthermore, high-quality studies are needed to unify the cut-off points of ESVEA so that we can monitor the occurrence of ESVEA in the clinic. Finally, we must conduct clinical trials of ESVEA interventions for the prevention of atrial fibrillation or stroke.

## 5. Conclusions

In conclusion, ESVEA is associated with an increased risk of incident AF, new-onset ischemic stroke, and all-cause mortality. Larger and more rigorous studies are urgently needed in the future to verify the relationship between ESVEA and ischemic stroke.

## Figures and Tables

**Table 1:** Characteristics of the included studies.

Study	Country	Study Design	Population	Number of Subjects	Age (Means ± SD)	Male n (%)	Concomitant Diseases (%)	Follow-up
Binici 2010 [12]	Denmark	Prospective cohort	Without CVD, stroke, or AF	678	64.5 ± 6.8	397 (58.6)	Diabetes (11.1)	6.3 years
Chong 2012 [13]	China	Prospective cohort	Without AF or structural heart disease	428	66.7 ± 10.2	187 (43.7)	Hypertension (45.3) Diabetes (17.1) CVD (17.5)	6.1 years
Dewland 2013 [14]	United States	Prospective cohort	Without prevalent AF	1260	71	569 (45)	Hypertension (55) Diabetes (15) CVD (20)	13.0 years
Yodogawa 2013 [33]	Japan	Prospective cohort	With AIS, without a history of AF	68	69.9 ± 9.6	37 (54.4)	Hypertension (66.2) Diabetes (14.7)	11 ± 4 months
Pinho 2015 [42]	Portugal	Retrospective cohort	With CIS or TIA	184	55.2 ± 15.1	96 (52.2)	Hypertension (56.5) Diabetes (14.7) Dyslipidemia (72.8) CVD (3.3)	27.5 months
Acharya 2015 [15]	United States	Retrospective cohort	Free of AF	1357	64	1262 (93)	Hypertension (66) Diabetes (22.6) CVD (19.7)	7.5 years
Johnson 2015 [34]	Sweden	Prospective cohort	Free of AF	383	64.6 ± 5.9	172 (45)	-	10.3 years
Lin 2015 [43]	China	Retrospective cohort	Without AF and a PPM	5371	61.8 ± 18.6	3222 (60)	Hypertension (35.6) Diabetes (20.2) Dyslipidemia (12.8) CVD (29.4)	10 ± 1 years
Larsen 2015 [23]	Denmark	Prospective cohort	Without CVD, stroke, or AF	678	64.5 ± 6.8	397 (58.6)	Diabetes (11.1)	14.4 years
Vinther 2016 [22]	Denmark	Retrospective cohort	With IS and without known AF	565	-	313 (55.4)	Hypertension (41.9) Diabetes (10.8)	4 years
Cabrera 2016 [44]	Spain	Retrospective cohort	Free of AF	299	62.5 ± 17.9	160 (53.5)	Hypertension (52.3) Diabetes (17.4)	39.1 months
Marinheiro 2017 [35]	Portugal	Prospective cohort	Without stroke or AF	362	-	204 (56.4)	Hypertension (77.6) Diabetes (25.1)	7.1 years
Vinther 2017 [36]	Denmark	Prospective cohort	With AIS and without AF	256	73 ± 12.6	141 (55)	Hypertension (57) Diabetes (13) Dyslipidemia (28) CVD (13)	32 months
Raman 2017 [37]	United States	Prospective cohort	Without baseline AF	2350	75.8 ± 5.3	2350 (100)	Hypertension (49) Diabetes (13.1)	8.0 ± 2.6 years
Persson 2019 [38]	Sweden	Prospective cohort	Free of AF	377	65 ± 6	170 (45)	-	17 years
Ntaios 2020 [39]	United Kingdom	Prospective cohort	Embolic Stroke of Undetermined Source	853	67	486 (57)	Hypertension (61.9) Diabetes (18.5) CVD (15)	3.4 years
Sejr 2020 [40]	Denmark	Prospective cohort	with AIS or TIA and without AF	1453	72.8 ± 7.7	822 (56.6)	Hypertension (58.6) Diabetes (14.3)	2.3 ± 1.3 years
Hygrell 2021 [45]	Sweden	Retrospective cohort	Free of AF	6100	76	2755 (45)	Hypertension (28) Diabetes (10)	4.2 years
Sasaki 2021 [46]	Japan	Retrospective cohort	Free of AF	138	72 ± 10	108 (52)	Hypertension (62.3) Diabetes (23.9) Dyslipidemia (39.1)	5 years
Vetta 2022 [41]	Italy	Prospective cohort	With cryptogenic stroke	112	72.2 ± 12.2	65 (58)	Hypertension (81) Diabetes (21) CVD (9)	6 months

CVD—cardiovascular disease; AF—atrial fibrillation; IS—ischemic stroke; AIS—acute ischemic stroke; CIS—cryptogenic ischemic stroke; TIA—transient ischemic attack; PPM—permanent pacemaker.

**Table 2:** Characteristics of ESVEA definition, ESVEA prevalence, and outcomes.

Study	Definition of ESVEA	Detection of ESVEA	Prevalence of ESVEA n (%)	Definition of Outcome	Numbers of Outcome n (%)
Binici 2010 [12]	≥30 SVEC/h or any episode of runs of ≥20 SVEC	48 h ECG	99 (14.6)	AF IS All-cause mortality	22 (5.5) 27 (6.7) 87 (21.4)
Chong 2012 [13]	>100 PACs/24 h	24 h ECG	107 (25)	AF IS Death	60 (14) 41 (9.6) 60 (14)
Dewland 2013 [14]	The median PAC count was 2.5 beats/h (IQR, 0.8 to 9.5 beats/h)	24 h ECG	-	AF All-cause mortality	343 (27.2) 837 (66.4)
Yodogawa 2013 [33]	>100 PACs/24 h	24 h ECG	-	AF	17 (25)
Pinho 2015 [42]	>30 APCs/h	24 h ECG	17 (9.2)	Recurrent IS/TIA	22 (12)
Acharya 2015 [15]	≥100 PACs/24 h	24 h ECG	486 (35.8)	AF	155 (11.4)
Johnson 2015 [34]	30 SVE/h and/or any SVT lasting for ≥20 consecutive beats	24 h ECG	-	AF	45 (11.7)
Lin 2015 [43]	PAC burden >76 beats per day	24 h ECG	2072 (38.6)	All-cause mortality AF	1209 (22.5) 418 (7.8)
Larsen 2015 [23]	≥30 PACs/h or any episode of runs of ≥20 PACs	48 h ECG	99 (14.6)	IS All-cause mortality	73 (10.8) 259 (38.2)
Vinther 2016 [22]	≥3 PACs lasting less than 30 s during 48 h	48 h of CICT	161 (28)	Recurrent IS/TIA All-cause mortality AF	73 (12.9) 158 (28) 22 (3.9)
Cabrera 2016 [44]	Percentage of PAC (during the 24 h period) ≥0.2%	24 h ECG	-	AF	31 (10.4)
Marinheiro 2017 [35]	>97 PACs/h	24 h ECG	124 (34.3)	IS All-cause mortality	54 (14.9) 129 (35.6)
	30–97 PACs/h		114 (31.5)		
Vinther 2017 [36]	>14 PACs/h and ≥3 runs of PACs/24 h	24 h ECG	31 (12.1)	Recurrent stroke All-cause mortality	20 (7.8) 34 (13.3)
Raman 2017 [37]	PAC/h of sleep ≥ 21.15	PSG based ECG	-	AF	269 (11.4)
Persson 2019 [38]	Top quartile of PACs (≥5.5 per hour) or SVT (≥0.13 per hour)	24 h ECG	-	AF	80 (21.2)
Ntaios 2020 [39]	>0–1 SVE per 10 s	12-lead ECG	111 (13)	AF Recurrent IS All-cause mortality	125 (14.7) 103 (12.1) 149 (17.5)
	>1–2 SVE per 10 s		57 (6.7)		
	>2 SVE per 10 s		58 (6.8)		
Sejr 2020 [40]	0.5–1.5 PACs/h	48 h ECG	359 (24.7)	AF Recurrent IS Recurrent TIA All-cause mortality	44 (2.9) 78 (5.4) 76 (5.2) 123 (8.5)
	1.6–5.9 PACs/h		365 (25.1)		
	≥6.0 PACs/h		362 (24.9)		
Hygrell 2021 [45]	the top tenth percentile according to SVECs count	30 s ECG	709 (11.6)	AF IS Death	387 (6.3) 161 (2.6) 354 (5.8)
Sasaki 2021 [46]	PAC burden ≥ 0.4%	24 h ECG	-	AF	61 (29.3)
Vetta 2022 [41]	PACs burden ≥ 7	24 h ECG	-	AF	24 (21.4)

SVEC—supraventricular ectopic complexes; AF—atrial fibrillation; ECG—electrocardiography; PACs—premature atrial complexes; IQR—interquartile range; APCs—atrial premature complexes; IS—ischemic stroke; TIA—transient ischemic attack; SVE—supraventricular extrasystoles; SVT—supraventricular tachycardias; CICT—continuous inpatient cardiac telemetry; SVECs—supraventricular ectopic complexes; PSG—polysomnogram.

## Data Availability

Not applicable.

## Supplementary Materials

The following supporting information can be downloaded at: https://www.mdpi.com/article/10.3390/jcdd9120461/s1, Figure S1: The funnel plot for estimating the association between ESVEA and the risk of AF in the general population, Figure S2: The funnel plot for estimating the association between ESVEA and the risk of all-cause mortality, Figure S3: Forest plot of the association between ESVEA and new-onset ischemic stroke by subgroups, Figure S4: Forest plot of the association between ESVEA and recurrent ischemic stroke/transient ischemic attack (TIA) by subgroups; Table S1: Quality assessment for the included studies.
