# Supplementary material for: Excessive Supraventricular Ectopic Activity and the Risk of Atrial Fibrillation and Stroke: A Systematic Review and Meta-Analysis
Source: J Cardiovasc Dev Dis. 2022 Dec 15;9(12):461. doi: 10.3390/jcdd9120461 (PMC9784080; doi:10.3390/jcdd9120461)
Supplement: Supplementary file 1 [file jcdd-09-00461-s001.zip › jcdd-2083955-supplementary.pdf]

Search strategy

pubmed

/*atrial premature complexes*/

1. atrial premature complexes OR premature supraventricular beat OR premature atrial beat OR premature atrial complex OR premature atrial contraction OR atrial ectopic beat OR atrial extrasystole

/*stroke*/

2. stroke OR cerebrovascular accident OR cerebrovascular apoplexy OR brain vascular accident OR apoplexy

/*atrial fibrillation*/

3. atrial fibrillation
4. 2 or 3

5. 1 and 4

embase

/*atrial premature complexes*/

1. exp supraventricular premature beat/ or (atrial premature complexes or premature supraventricular beat or premature atrial beat or premature atrial complex or premature atrial contraction or atrial ectopic beat or atrial extrasystole).mp.

/*stroke*/

2. exp cerebrovascular accident/ or (stroke or cerebrovascular apoplexy or brain vascular accident or apoplexy).mp.

/*atrial fibrillation*/

3. exp atrial fibrillation/
4. 2 or 3

5. 1 and 4

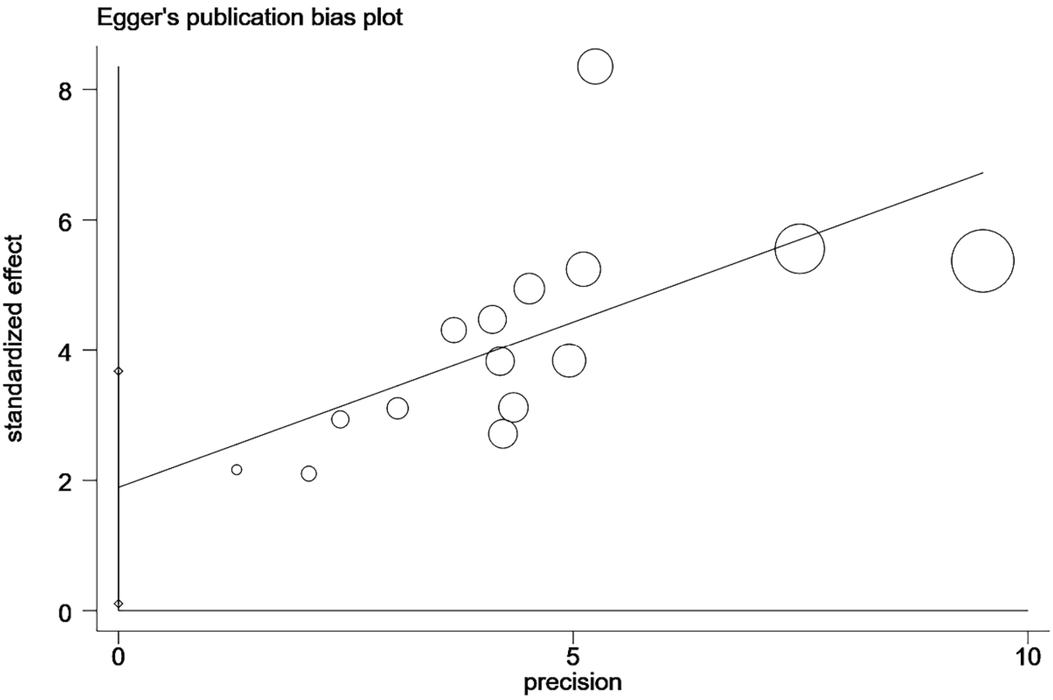

Figure S1. The funnel plot for estimating the association between ESVEA and the risk of AF in the general population.

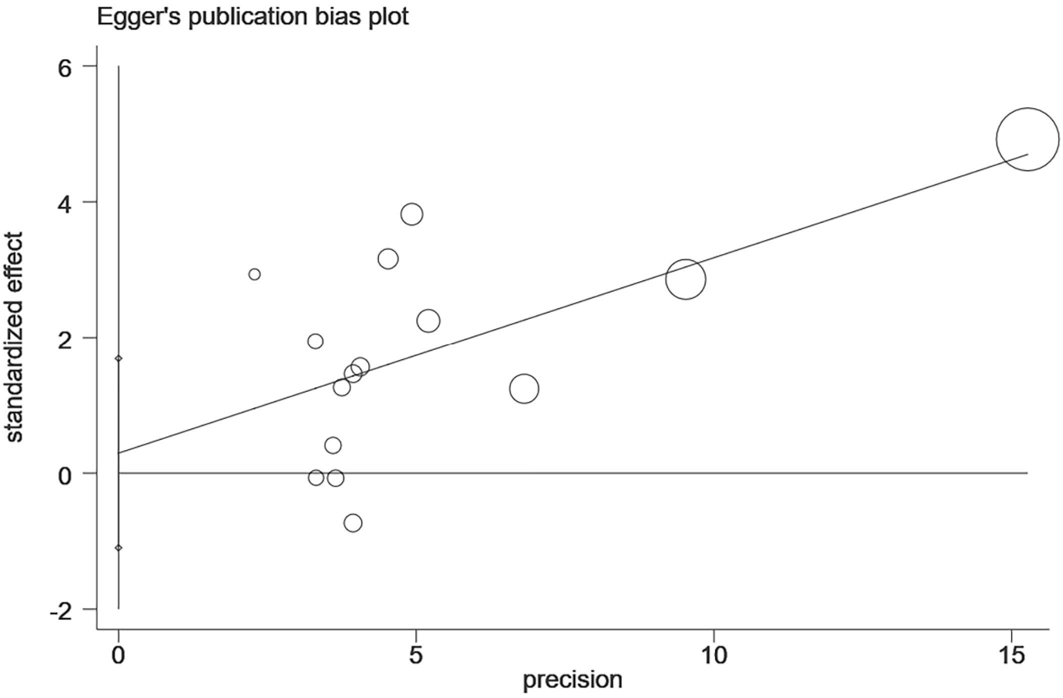

Figure S2. The funnel plot for estimating the association between ESVEA and the risk of all-cause mortality.

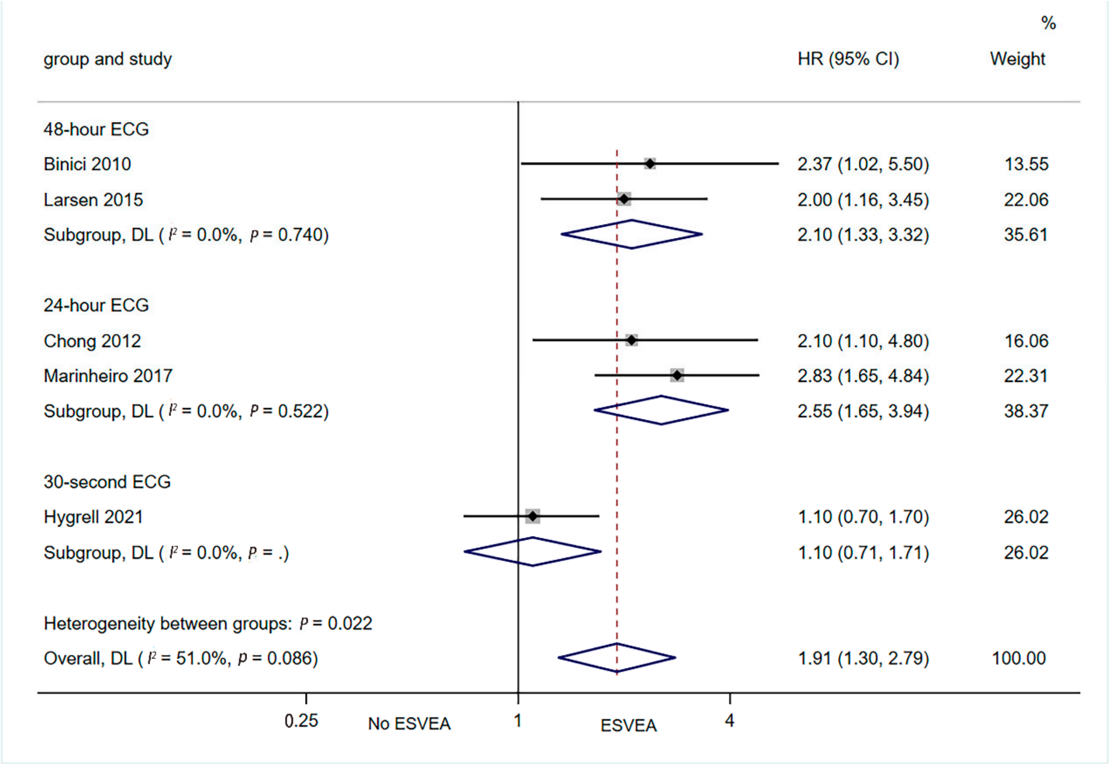

Figure S3. Forest plot of the association between ESVEA and new-onset ischemic stroke by subgroups. Abbreviations: ESVEA, excessive supraventricular ectopic activity; HR, hazard ratio; CI, confidence interval. [12, 23, 13, 35, 45]

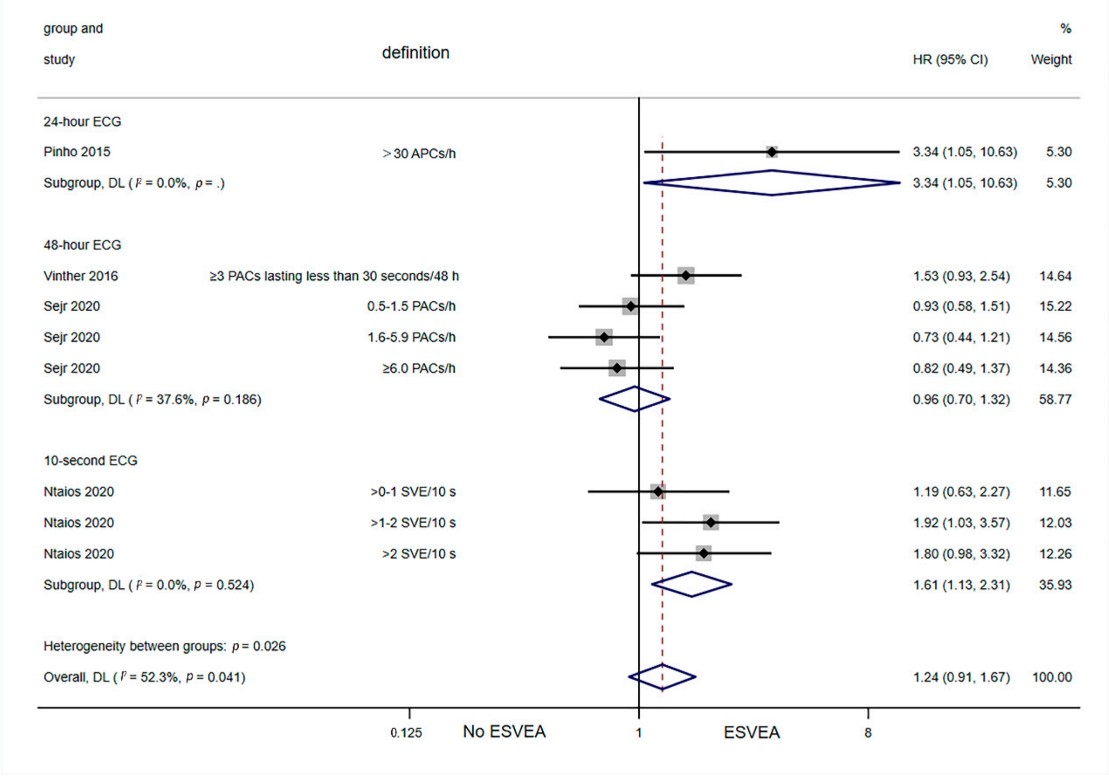

Figure S4. Forest plot of the association between ESVEA and recurrent ischemic stroke/transient ischemic attack (TIA) by subgroups. Abbreviations: APCs, atrial premature complexes; PACs, premature atrial complexes; SVE, supraventricular extrasystoles; ESVEA, excessive supraventricular ectopic activity; HR, hazard ratio; CI, confidence interval. Definition represents the definitions of ESVEA in different studies. [42, 22, 40, 39]

Table S1. Quality assessment for the included studies.

Study	Study type	Selection				Comparability	Outcome			Total scale
Binici 2010 [12]	Cohort	★	★	★	★	★★	★	★	-	8
Chong 2012 [13]	Cohort	★	★	★	★	★★	★	★	-	8
Dewland 2013 [14]	Cohort	★	★	★	★	★★	★	★	-	8
Yodogawa 2013 [33]	Cohort	★	★	★	★	★★	★	★	-	8
Pinho 2015 [42]	Cohort	★	★	★	-	★★	★	★	-	7
Acharya 2015 [15]	Cohort	★	★	★	-	★★	★	★	-	7
Johnson 2015 [34]	Cohort	★	★	★	★	★★	★	★	-	8
Lin 2015 [43]	Cohort	★	★	★	-	★★	★	★	-	7
Larsen 2015 [23]	Cohort	★	★	★	★	★★	★	★	★	9
Vinther 2016 [22]	Cohort	★	★	★	-	★★	★	★	★	8
Cabrera 2016 [44]	Cohort	★	★	★	-	★★	★	★	★	8
Marinheiro 2017 [35]	Cohort	★	★	★	★	★★	★	★	★	9
Vinther 2017 [36]	Cohort	★	★	★	-	★★	★	★	★	8
Raman 2017 [37]	Cohort	★	★	★	★	★★	★	★	-	8
Persson 2019 [38]	Cohort	★	★	★	★	★★	★	★	★	9
Ntaios 2020 [39]	Cohort	★	★	★	-	★★	★	★	-	7

Sejr 2020 [40]	Cohort	★	★	★	-	★★	★	★	-	7
Hygrell 2021 [45]	Cohort	★	★	★	-	★★	★	★	★	8
Sasaki 2021 [46]	Cohort	★	★	★	-	★★	★	★	-	7
Vetta 2022 [41]	Cohort	★	★	★	★	★★	★	★	-	8

One "★" represents one point; "-" represents no points
